# Genetic Architecture of Soybean Yield and Agronomic Traits

**DOI:** 10.1534/g3.118.200332

**Published:** 2018-08-28

**Authors:** Brian W. Diers, Jim Specht, Katy Martin Rainey, Perry Cregan, Qijian Song, Vishnu Ramasubramanian, George Graef, Randall Nelson, William Schapaugh, Dechun Wang, Grover Shannon, Leah McHale, Stella K. Kantartzi, Alencar Xavier, Rouf Mian, Robert M. Stupar, Jean-Michel Michno, Yong-Qiang Charles An, Wolfgang Goettel, Russell Ward, Carolyn Fox, Alexander E. Lipka, David Hyten, Troy Cary, William D. Beavis

**Affiliations:** * Department of Crop Sciences, University of Illinois, Urbana, IL, 61801; † Department of Agronomy and Horticulture, University of Nebraska, Lincoln, NE, 68583; ‡ Department of Agronomy, Purdue University, West Lafayette, IN, 47907; § USDA-ARS, Beltsville, MD, 20705; ** Department of Agronomy, Iowa State University, Ames, IA, 50011; †† USDA-ARS and Department of Crop Sciences, University of Illinois, Urbana, IL, 61801; ‡‡ Department of Agronomy, Kansas State University, Manhattan, KS, 66506; §§ Department of Plant, Soil and Microbial Sciences, Michigan State University, East Lansing, MI, 48824; *** Division of Plant Sciences, University of Missouri Delta Center, Portageville, MO, 63873; ††† Department of Horticulture and Crop Science, The Ohio State University, Columbus, OH, 43210; ‡‡‡ Plant, Soil, and Agricultural Systems, Southern Illinois University, Carbondale, IL, 62901; §§§ Dow AgroSciences, Indianapolis, IN, 46268; **** USDA-ARS, Raleigh, NC, 27606; †††† Department of Agronomy and Plant Genetics, University of Minnesota, St. Paul, MN, 55108; ‡‡‡‡ USDA-ARS Plant Genetic Research Unit at Donald Danforth Plant Science Center, St. Louis, MO, 63132

**Keywords:** soybean, genetic improvement, yield, genetic mapping, Multiparent Advanced Generation Inter-Cross (MAGIC), multiparental populations, MPP

## Abstract

Soybean is the world’s leading source of vegetable protein and demand for its seed continues to grow. Breeders have successfully increased soybean yield, but the genetic architecture of yield and key agronomic traits is poorly understood. We developed a 40-mating soybean nested association mapping (NAM) population of 5,600 inbred lines that were characterized by single nucleotide polymorphism (SNP) markers and six agronomic traits in field trials in 22 environments. Analysis of the yield, agronomic, and SNP data revealed 23 significant marker-trait associations for yield, 19 for maturity, 15 for plant height, 17 for plant lodging, and 29 for seed mass. A higher frequency of estimated positive yield alleles was evident from elite founder parents than from exotic founders, although unique desirable alleles from the exotic group were identified, demonstrating the value of expanding the genetic base of US soybean breeding.

Soybean is a leading source of vegetable protein and oil worldwide. Of all major crops, soybean showed the greatest annual increase in global production area during the past 40 years (Hartman *et al.* 2011). In the 2015/2016 production season, it was grown on 119.7 million hectares worldwide, producing 313.2 million metric tons of grain (USDA 2016). A nonlinear regression analysis of the 1924-2012 USA soybean yield trajectory revealed an annual increase of 21.5 kg ha^-1^ prior to 1983 and 29.4 kg ha^-1^ thereafter, with *ca*. 2/3 of the recent annual yield gains attributable to improvements in genetic yield potential (Specht *et al.* 2014). To meet expectations of greater future demand for soybean, the current improvement rate needs to double to avoid increasing the production area (Ray *et al.* 2013).

Genetic improvement in soybean yield potential has been achieved by mating selected homozygous lines, deriving segregating progeny through self-pollination to create replicable lines and assessing the performance of lines in multiple years of field trials to select those that will be mated for the next cycle (Bernardo 2002). This iterative method has been successful, despite a limited understanding of the genomics of key physiological mechanisms, whose phenotypic/genotypic variance underpins much of the variance in yield. Phenology is critical because greater yield can be positively correlated with later maturity in soybean. Of the mapped quantitative trait loci (QTL) listed in SoyBase (Grant *et al.* 2010), there are 79 yield and 82 maturity non-redundant QTL, but 40 of each are closely linked (<5 to 0 cM) or possibly pleiotropic. Though QTL mapping is relatively powerful for identifying associations between segregating alleles and phenotypes, it cannot provide high-resolution map positions when recombination events are rare.

Only a subset of the total number of QTL affecting a trait can be detected when using segregating lines derived from a single cross between two parents. Alternatively, greater map resolution of marker-trait associations (MTAs) can be achieved using genome-wide association studies (GWAS), which exploit historical recombination events. However, GWAS is not as powerful as QTL linkage mapping (Hirschhorn and Daly 2005; Kingsmore *et al.* 2008), nor can it detect rare alleles (even those of large effect), as documented in both theory (Korte and Farlow 2013) and practice (Bandillo *et al.* 2015). In contrast, the opportunity to detect rare alleles increases when QTL mapping is applied to scores of bi-parental matings, and when the lines used for initial crosses have been purposely selected to be phenotypically diverse (Phansak *et al.* 2016).

Nested association mapping (NAM) takes advantage of both linkage and association mapping to increase map resolution and statistical power (Yu *et al.* 2008). In the NAM design, a common inbred parent is mated to a diverse group of homozygous founders to create thousands of recombinant inbred lines (RILs) in half-sib families. In the maize NAM project, the inbred B73 was crossed with 25 distinct lines adapted to a wide range of latitudes from the tropics to Canada (Buckler *et al.* 2009; McMullen *et al.* 2009). The maize NAM families have been used to map QTL for several traits including disease resistance (Kump *et al.* 2011; Poland *et al.* 2011; Olukolu *et al.* 2014) flowering time (Buckler *et al.* 2009), kernel composition (Cook *et al.* 2012) and plant type and architecture (Tian *et al.* 2011; Peiffer *et al.* 2013; Brown *et al.* 2011). The NAM population structure also was used to map QTL for stem rust in wheat (Bajgain *et al.* 2016), and flowering time and grain weight in barley (Maurer *et al.* 2015; Maurer *et al.* 2016). The objectives of our project were to map marker-trait associations for yield and other important agronomic traits in a soybean NAM population with a goal of improving our understanding of the genetic basis of these traits and to identify exotic sources of genes that can improve yield. To our knowledge, no prior NAM populations have been used to study the genetic architecture of grain yield, the most important trait for most major field crops.

## Materials and Methods

### Germplasm development

The photoperiod sensitivity of soybean forces breeders to develop cultivars and lines adapted to specific latitudes. To minimize the confounding effect of non-adaptation in the soybean NAM population developed for this study, 40 soybean founder lines were selected for adaption to the major North American soybean production region occupying a latitudinal zone of *ca*. 37° to 43° N, and were mated to the high-yielding common parent IA3023, which is adapted to *ca*. 40° N. By limiting the latitude adaptation of the parents, we were able to develop recombinant inbred lines that could be evaluated for agronomic traits in common environments to reduce the confounding effect of photoperiod responses on these traits. Many potential founders were initially nominated by soybean breeders and a limited SNP-based diversity analysis of these nominees was used to identify a final set of 40 founders (Song *et al.* 2017). These consisted of 17 high-yielding elite cultivars and breeding lines (EL), 15 breeding lines selected for yield and exotic diversity (BX), and eight plant introductions (PI) that yielded well under severe drought in field trials (Song *et al.* 2017) (Table 1). From each of the 40 matings, 140 RILs were derived using single-seed descent from the F_2_ to F_5_ generation, resulting in 5,600 RILs.

**Table 1:** Founders of the 40 NAM families, their origin and group. For more information and photos, see: https://soybase.org/SoyNAM/imagebrowser.php

NAM Family	Parent	Origin	Group*
Hub	IA3023	Iowa State Univ.	Common parent
N02	TN05-3027	Univ. of Tenn.	EL
N03	4J105-3-4	Purdue Univ.	EL
N04	5M20-2-5-2	Purdue Univ.	EL
N05	CL0J095-4-6	Purdue Univ.	EL
N06	CL0J173-6-8	Purdue Univ.	EL
N08	HS6-3976	Ohio State Univ.	EL
N9	Prohio	USDA-ARS, Wooster, OH	EL
N10	LD00-3309	Univ. of Illinois	EL
N11	LD01-5907	Univ. of Illinois	EL
N12	LD02-4485	Univ. of Illinois	EL
N13	LD02-9050	Univ. of Illinois	EL
N14	Magellan	Univ. of Missouri	EL
N15	Maverick	Univ. of Missouri	EL
N17	S06-13640	Univ. of Missouri	EL
N18	NE3001	Univ. of Nebraska	EL
N22	Skylla	Mich. State Univ.	EL
N23	U03-100612	Univ. of Nebraska	EL
N24	LG03-2979	USDA-ARS, Urbana, IL	BX
N25	LG03-3191	USDA-ARS, Urbana, IL	BX
N26	LG04-4717	USDA-ARS, Urbana, IL	BX
N27	LG05-4292	USDA-ARS, Urbana, IL	BX
N28	LG05-4317	USDA-ARS, Urbana, IL	BX
N29	LG05-4464	USDA-ARS, Urbana, IL	BX
N30	LG05-4832	USDA-ARS, Urbana, IL	BX
N31	LG90-2550	USDA-ARS, Urbana, IL	BX
N32	LG92-1255	USDA-ARS, Urbana, IL	BX
N33	LG94-1128	USDA-ARS, Urbana, IL	BX
N34	LG94-1906	USDA-ARS, Urbana, IL	BX
N36	LG97-7012	USDA-ARS, Urbana, IL	BX
N37	LG98-1605	USDA-ARS, Urbana, IL	BX
N38	LG00-3372	USDA-ARS, Urbana, IL	BX
N39	LG04-6000	USDA-ARS, Urbana, IL	BX
N40	PI 398.881	South Korea	PI
N41	PI 427136	South Korea	PI
N42	PI 437169B	Russia	PI
N46	PI 507681B	Uzbekistan	PI
N48	PI 518751	Serbia	PI
N50	PI 561370	China	PI
N54	PI 404188A	China	PI
N64	PI 574486	China	PI

* Founder group designations are EL = Elite, BX = breeding lines with exotic ancestry, and PI = plant introduction.

### Field evaluation

The common parent and founder lines, their RILs and check cultivars were grown in two-row field plots (0.76 m spacing; *ca*. 4 m long) replicated across 22 environments in eight Midwestern USA states from 2011 to 2013. RILs from each family were sub-divided into four sets of 35, with each set augmented with the family’s two parents and three check cultivars chosen for adaptation to the field environments. If sufficient seed was not available for a RIL entry, the plot was planted with a check variety. The entries within each set were randomized and the 40-entry sets were randomized within field sites. All 5,600 RILs (160 sets) were evaluated at eight sites, while at the remaining sites only 25 to 100 sets, with an average of 50 sets, were evaluated due to capacity limitations of the cooperators (Table 2).

**Table 2:** The number of field plots, environments and blocks used to obtain BLUP values for the agronomic traits, estimates of broad-sense heritability (H) on an entry mean basis with 95% confidence intervals (CI), and the proportion of genotypic variance attributable to variance among families (σ^2^_g_ among families)

Trait	Number of plots	Number of environments (and field Blocks)	H (95% CI)	σ^2^_g_ among families
Yield (kg ha^-1^)	66,684	17 (1726)	0.822 (0.815, 0.829)	0.57
Maturity (days)	58,714	15 (1541)	0.935 (0.932, 0.937)	0.25
Lodging (1-5 scale)	57,420	15 (1351)	0.824 (0.817, 0.831)	0.18
Plant height (cm)	57,822	15 (1490)	0.938 (0.936, 0.941)	0.28
Seed mass [g (100 seed)^-1^]	52,703	10 (1222)	0.939 (0.936, 0.941)	0.26

The plot-combined seed weight and seed moisture content were measured electronically, and these data were used to calculate seed yield (kg ha^-1^ on a 130 g kg^-1^ moisture basis). Plant maturity was rated as days from planting to stage R8 (95% of pods fully mature), plant height as the cm distance from the soil surface to the top node on the main stem, plant lodging score rated visually on a scale of 1 = all plants erect to 5 = all plants prostrate and the yield component of seed mass as g (100 seed)^-1^. Any plot data discarded by the cooperating scientist who judged that data to be of poor quality were treated as missing data. The number of plots evaluated for each trait ranged from 52,000 to over 66,000 (Table 2).

### Marker evaluation

NAM RILs were genotyped with SNP markers (Song *et al.* 2016). Briefly, the common parent and 40 founder lines were sequenced with an Illumina HiSeq 2000 to identify SNP loci segregating in at least 28 (70%) of the 40 families based on marker allele differences between the common and founder lines. A total of 6,000 SNPs were selected from those that met this criterion and were submitted to Illumina, which identified 5,303 that were capable of associating with bead types on a BeadChip designed for the project (SoyNAM6K). The chip successfully detected 4,312 SNPs in the NAM parents and RILs. Markers that were non-polymorphic, or exhibited severe segregation distortion (*i.e.*, a minor allele frequency of less than 10%), in any family were eliminated, resulting in 2,470 to 3,791 SNP loci segregating within individual families.

The NAM common parent and founder lines (but not RILs) were evaluated with the SoySNP50K BeadChip (Song *et al.* 2013) that detected the segregation of 42,509 SNP markers among these parents. The framework of the mapped SoyNAM6K markers was then used to project the segregating SoySNP50K markers onto the NAM RILs using the Williams 82 reference genome (Wm82.a2.v1) bp positions for both the 6K and 50K chip SNP markers (Tian *et al.* 2011). The combined dataset of SoyNAM6K and SoySNP50K markers were then used to identify MTAs throughout the genome.

Linkage mapping was not conducted for the SoySNP50K SNP markers with the SoyNAM RILs. Instead, estimated cM linkage map positions of the SNPs were established using a linkage map derived from a Williams 82 x *G. soja* PI 479752 (WxP) population of 1083 RILs genotyped with 21,000 SoySNP50K SNPs (Song *et al.* 2016). The genetic location (cM) of any NAM SNP not present in the WxP map was inferred by linear interpolation between the NAM SNP physical position relative to the physical positions of the flanking WxP mapped SoySNP50K SNPs.

An initial analysis of the SNP-genotyped RILs was conducted to identify RILs that deviated from the expected marker segregation (Song *et al.* 2017), and this led to 424 RILs being discarded because they had a SNP genotype identical with the female founder (*i.e.*, were likely inadvertent female-parent self-pollinations), or they segregated for alleles that did not match the parent alleles. Most of the RILs from family N46 (PI507618B) fell into the latter category, indicating that a line other than the intended founder PI had been used in the mating with IA3023. Therefore, all lines from the N46 family were removed from the dataset.

### Data analyses

#### Agronomic phenotypes:

Field plots were planted when conditions were suitable. Due to protracted and excessive rain some environments experienced late planting. Planting dates spanned four weeks in both 2012 and 2013. Therefore, Julian planting dates were used as a covariate in the analyses of agronomic trait values. In addition, environmental conditions due to soils, pests and diseases were inconsistent between years, locations and even within location-year combinations. Incomplete blocks within environments were augmented with IA3023 and at least three additional check varieties to provide estimates of block effects for purposes of adjusting genotypic values for agronomic traits. Two check varieties, IA3023 and U06-100052 were included in all blocks, two were included in 77.5% of the blocks and the remaining six checks were unevenly distributed among the blocks. Due to the imbalance of check varieties assigned to blocks as well as the variable number of blocks evaluated among environments, we used shrunken, *i.e.*, best linear unbiased predicted values, rather than average values for field block effects that were based on the mixed linear model:

$$y=X\beta \mathrm{+ C}\kappa \mathrm{+ B}\pi \mathrm{+ \varepsilon},\pi ∼N(\mathrm{0,I}\sigma_{blk}^{2}),\varepsilon ∼N(\mathrm{0,I}\sigma_{res}^{2}),\text{Cov}(\pi ,\varepsilon )=0$$ (1)

For yield, kg/ha, **y** consisted of a vector of 7537 values for ten check varieties evaluated in 1726 blocks, **X** is a vector of planting dates for the checks in each block and is treated as a fixed effect, **β** is the slope and intercept for the covariate, planting date, **C** represents an incidence matrix for the check varieties, **κ** is the vector of fixed effects represented by the check varieties, **B** is an incidence matrix indicating whether the **y** value was obtained from a block, **π** is the vector of random effects represented by each block, **I** is the identity matrix, $\sigma_{blk}^{2}$ is the variance among blocks, **ε** represents a residual effect, not accounted for in the model, and $\sigma_{res}^{2}$is the variance among residual values. Best Linear Unbiased Estimates (BLUE’s) of slope and intercept for planting date and the check variety values as well as BLUP values for block effects were obtained using the lmer package in R (Bates *et al.* 2015).

RIL agronomic phenotypes were evaluated in an unbalanced design across environments. Most RILs were evaluated for yield in seven environments, while subsets of RILs were evaluated for agronomic traits in the remaining environments (Table 2). Yield data were not collected from some of the 22 planted environments due to data quality issues caused by environmental problems at field locations. For example, yield data were not collected at one if the eight environments that all RILs were planted because of damage from a hail storm. Due to the imbalance of RIL’s evaluated among environments and variable numbers of blocks within environments a mixed linear model was used to analyze agronomic traits among the non-check entries:

$$y=\mathrm{X\beta +Z\upsilon +\varepsilon ,}\beta =[\beta_{pldate},\beta_{blk}],\upsilon ∼N(\mathrm{0,I}\sigma_{g}^{2}),\varepsilon ∼N(\mathrm{0,I}\sigma_{res}^{2}),\text{Cov}(\upsilon ,\varepsilon )=0$$ (2)

where **y** is a vector of measured phenotypic trait values for entries consisting of RILs and their founder parents. The length of **y** as well as the dimensions of the matrices, **X** and **Z**, depend on the agronomic traits (Table 2). For yield, **y** consists of a vector of 66,684 values for RILs and their founder parents evaluated at 17 environments in 1726 blocks. **X** consists of two vectors representing covariates for planting date and the estimated shrunken block values obtained from (1). Both covariates are treated as fixed effects in (2) and **β** are the slopes and intercepts for planting date and block effects. **Z** is an incidence matrix for entries (RILs and founder lines) indicating whether the measured trait value, **y**, for the entry was evaluated in a block, and **υ** is the vector of random effects for entries, **I** is the identity matrix, $\sigma_{g}^{2}$is the genotypic variance among entries and **ε** represents the residual value, not accounted for in the model and $\sigma_{res}^{2}$is the variance among residual values.

Estimates of variance components for model (2) were obtained using the lmer package (Bates *et al.* 2015). Because the variance component estimates were from unbalanced numbers of environments, the estimated phenotypic variance was used to calculate broad sense heritability on both a family mean and an RIL entry mean basis. In these calculations, estimates of variance components were divided by the harmonic means for the number of environments in which the entries were evaluated (Holland *et al.* 2003). Harmonic means were likewise used to approximate confidence intervals for the estimated heritabilities (Knapp *et al.*, 1985).

#### GWAS analyses of agronomic phenotypes:

We used the BLUP genotypic values from model (2) for the analyses of genome wide associations in which we identified marker trait associations (MTAs) as random effects dependent on family background:

$$\upsilon =\mu +W\alpha +\varphi +\varepsilon ,\alpha ∼\text{N}(0,\text{I}\sigma_{\alpha }^{2}),\varphi ∼\text{N}(0,K\sigma_{\varphi }^{2}),\varepsilon ∼\text{N}(0,\text{I}\sigma_{\varepsilon }^{2}),\text{Cov}(\text{φ},\varepsilon )=0$$ (3)

Estimates of the model parameters were obtained using an empirical Bayes algorithm (Xavier *et al.* 2015) implemented in the R NAM package (https://CRAN.R-project.org/package=NAM). Genotypic values, **υ**, were BLUP values from (2). The polygenic term, **φ**, accounted for genetic structure among entries through the genomic relationship matrix, **K**. The model allowed each family, indicated by **W**, to have a unique estimated effect of allele substitution. The predicted values for allelic substitution, **α,** were interpreted as estimates of marker substitution effects from each of the founder lines. Because there were 39+1 founders, there were potentially 40 distinctive allelic substitution effects, conditioned by polygenic background (**φ**), at each of the marker loci. The polygene was estimated with the genomic relationship matrix **K** that captured the additive relationship among individuals, thereby accounting for population structure.

Implementation of (3) in the NAM package provided an option to exclude markers linked to the marker of interest from the polygenic background using a ‘linkage window’ (Xu and Atchley 1995). If this option is not used, then the estimated effects at each marker will be adjusted for polygenic background effects that include tightly linked markers, thus reducing power to detect significant associations. At the other extreme, if the linkage window is too large, then polygenic background effects will lead to false positive associations. For exploratory analyses of experimental data, it is unlikely that there is a single best size window. Based on the evaluation of several possible window sizes we chose to report results from a window size of 5 cM. MTAs for 5 cM regions were considered significant if the –log_10_(p-value) ≥ 3, which corresponds to a Bonferroni-corrected experiment-wide false positive probability of no greater than 0.1 for each trait. In effect, the window size of 5 cM enabled us to define unique genomic regions for multiple MTAs that were closely linked among segregating progeny in multiple families. GWAS with a window size of 20 cM resulted in a similar number of MTAs identified for yield (data not shown).

### Candidate gene identification

To identify candidate genes for the co-expression network analysis, plants of the NAM common and founder lines were grown in the Danforth Center Greenhouse (St Louis, MO) and seeds at the mid-maturation stage were harvested and used for purification of total RNA as previously described (Goettel *et al.* 2014). RNA-seq libraries were prepared with the TruSeq RNA Sample Preparation Kit v2 following the manufacture’s instruction (Illumina, Inc., San Diego, CA), and sequenced on the Illumina HiSeq2000. Sequence reads for each sample were independently aligned to the soybean reference genome (Wm82.a2.v1) (Schmutz *et al.* 2010) guided by the soybean gene annotation in Phytozome v10 using Tophat 2 (v2.0.10) (Kim *et al.* 2013). Cufflinks (v2.2.1) (Trapnell *et al.* 2012) was then run on each sample assembly to determine and normalize gene expression as the total fragments per kilobase of transcript per million mapped reads (FPKM).

To generate weighted co-expression networks, the NAM common and founder line expression matrix served as input into Camoco (Schaefer *et al.* 2018) with the following parameters; max_gene_missing_data = 0.5, max_accession_missing_data = 0.4, min_single_sample_expr = 1, min_expr = 0.001, and max_val = 300. The Camoco overlap command was used to identify genes enriched for density to other genes underlying MTA peaks using 1000 bootstraps. Fifteen different networks were generated using factorial combinations of interval sizes (within 10, 20, 50 100, and 500 kb of the most significant marker, respectively) and the number of genes surrounding the most significant marker (1, 2, and 5 flanking genes, respectively). Genes with a false discovery rate (FDR) less than 0.35 were deemed significant. Our decision criteria also required that a discovered gene be significant across at least two different parameters and have a direct connection to other candidate genes. Given the stringency of these filters, a more permissive FDR threshold of 0.35 was applied, similar to what Schaefer *et al.* (2018) previously used to successfully discover and validate a candidate gene in maize. Gene lists were filtered for genes that appeared as significant for more than one interval/flanking gene combination. Weighted edges for genes were transformed into binary variables, eliminating edges with a Z-score less than two. Genes that retained at least one connection after transforming the weights were further investigated.

### Data and germplasm availability

The quality-assured phenotypic and SNP genotypic data are available for download from SoyBase (https://soybase.org/SoyNAM/index.php). The site also contains forms for requesting seed samples of the NAM RILs and founder parents, and also images of the field-grown parents. Supplemental material available at Figshare: https://doi.org/10.25387/g3.6970496.

## Results and Discussion

**Figure 1:**
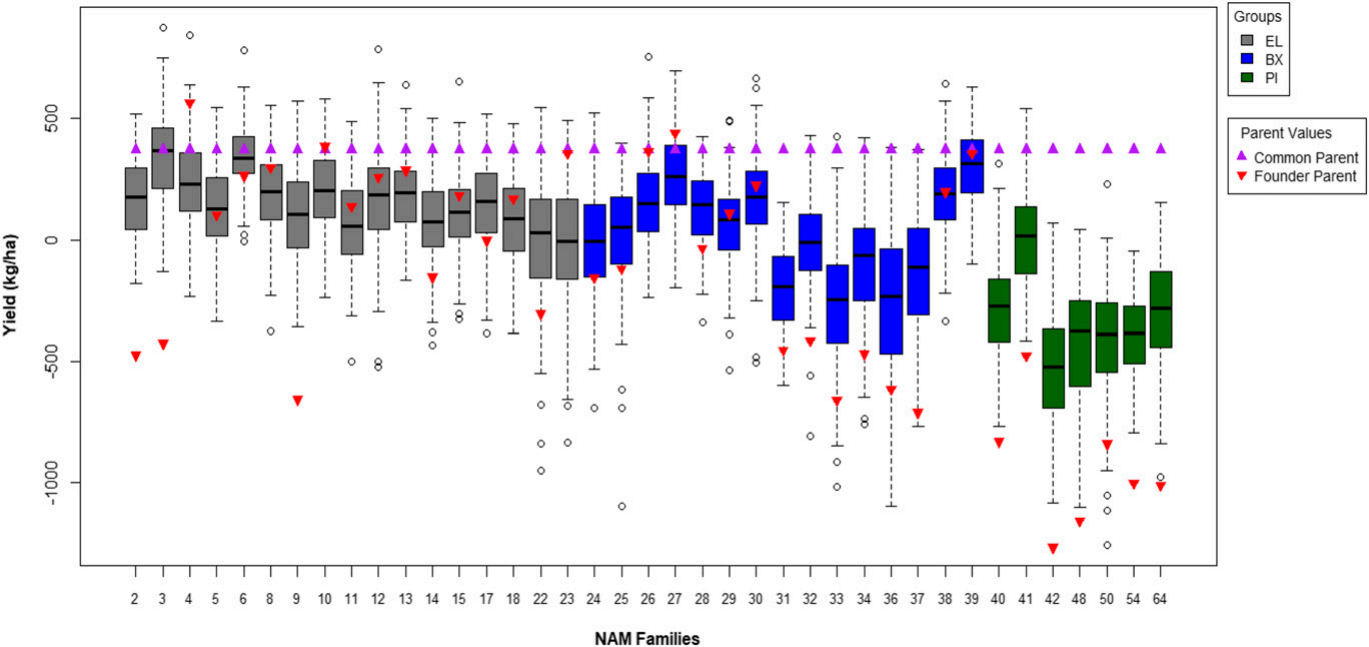
Box plots of best linear unbiased predicted (BLUP) values for yield in soybean NAM families developed from mating a common parent (IA3023, violet triangles) to 39 founders (red triangles) consisting of three parental group classes: EL = elite cultivars, BX = breeding lines with exotic ancestry and PI = plant introductions. The box spans interquartile range for each family, the horizontal line within each box denotes the median value, the capped dashed lines denote 95% span of RIL values, and the open circles denote values that exceed the 95% span. Founder parent names are listed on Table 1.

In a combined analysis of yield across 17 environments, the yield of the common parent IA3023 was superior to the yields of all founders except for N03 (4J105-3-4), N10 (LD00-3309) and N27 (LG05-4292) (Figure 1, Table 1). As expected, median family yields were uniformly greater for families descending from EL founders, generally lower for families from BX founders and even lower for families from PI founders.

Broad-sense heritability estimates for yield and agronomic traits on an entry mean (RIL) basis across families ranged from 0.82 to 0.93 (Table 2). Slightly more than half (0.57) of the yield genotypic variance among RILs was attributed to variance among family means while variance among RILs within families accounted for the remainder. For other agronomic traits, this proportion ranged from 0.18 to 0.28 (Table 2). Genotypic correlation estimates were 0.40 for yield and maturity, -0.20 for yield and lodging, but near-zero for yield and either plant height or seed mass.

**Figure 2:**
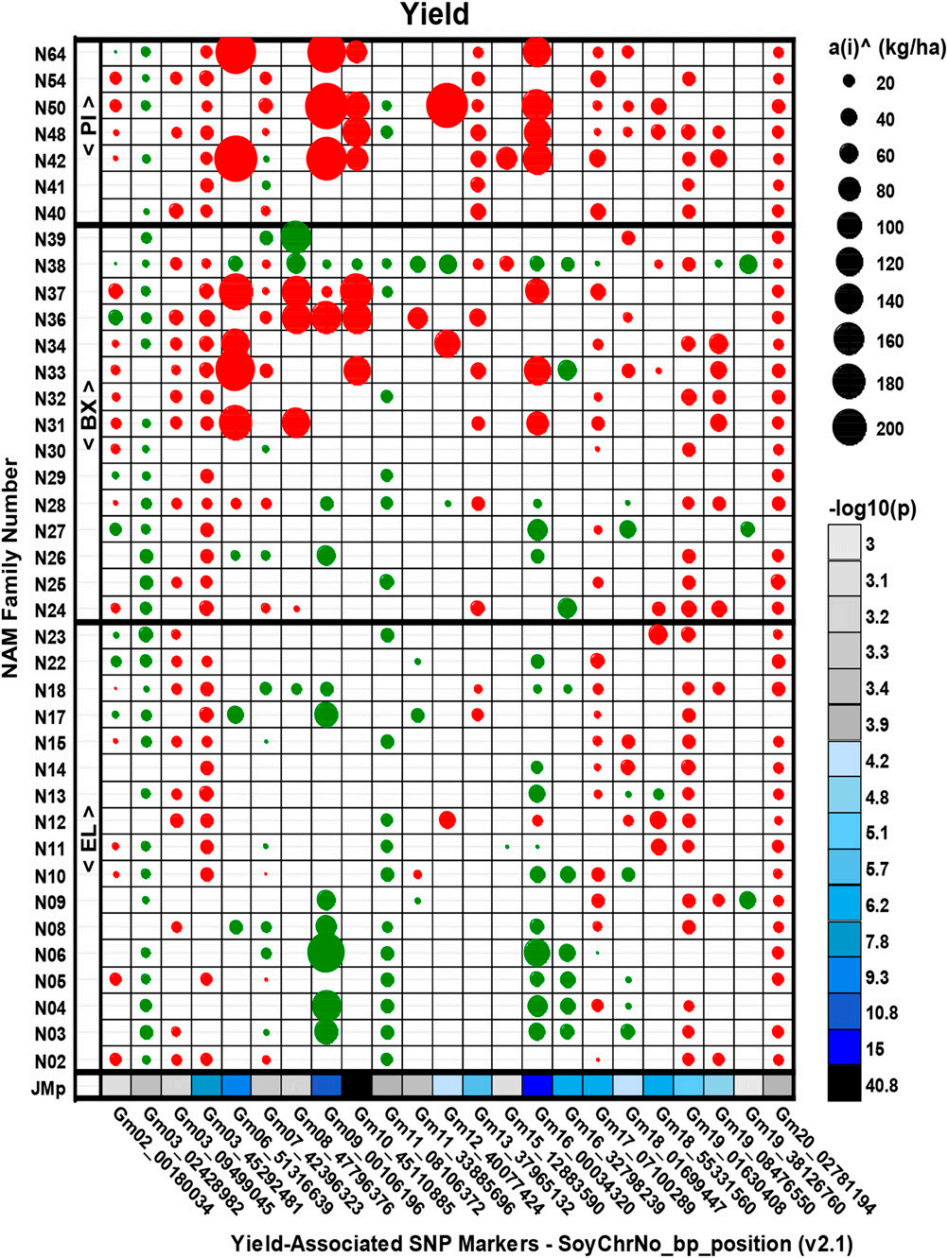
Marker trait associations for yield. The estimated magnitude of each allelic effect (in kg ha^-1^) is depicted by circle symbol diameter, with negative and positive effects relative to the common parent (IA3023) respectively depicted by red and green. The observed -log10(p) values for the each of the 23 marker-trait associations across the 39 founder families are column color-coded by magnitude in the row labeled JMp (acronym for Joint Mapping p value). Founder parent names are listed on Table 1.

A 5-cM linkage window size was used to distinguish multiple linked MTAs from broader background polygenic effects (Xu and Atchley 1995), and to establish distinct genomic regions containing linked MTAs for multiple traits (Table S1). Ultimately, 23 unique genomic regions on 16 chr were identified with statistically significant MTAs for yield (Figure 2), along with 19 regions on 16 chr for maturity, 15 on 12 chr for plant height, 17 on 10 chr for plant lodging, and 29 on 18 chr for seed mass (Figures S1-4).

Additive allelic effects for MTAs were estimated by family relative to IA3023. No allelic effects were estimable for any MTA-family combination for any trait in which the SNP marker was not segregating (Table S1; Figure 2; Figures S1-4). The number of families with additive effect alleles per MTA ranged from as few as one to as many as 38, and the allelic effects varied in sign and magnitude (Figure S5). For yield, negative allelic effects were detected in *all* founder families for *five* MTA regions (*i.e.*, two on chr 3, and one each on chr 13, 19, and 20), indicating that no founder allele was superior to the IA3023 allele at these loci (Figure 2). The allelic effect also was negative for *all but one* founder in *four* other regions (*i.e.*, chr 10, 15, 18, and 19) and for *all but two* in *one* other region (*i.e.*, chr 17). Conversely, a positive allelic effect was detected in *all* families in *four* significant MTA regions (*i.e.*, chr 3, 11, 16, and 19), implying that the yield potential of IA3023 might be improved by introgression of the corresponding estimated effect for the allele from founder parent alleles. The estimated allelic effects were split between the common allele and the founder alleles for the remaining *nine* MTAs, with negative allelic effects generally outnumbering positive allelic effects from the non-IA3023 founder, except for MTAs on chr 9 and 16. This implies that two different founder alleles might exist, with one being more, and the other less, favorable than the IA3023 allele. A sizeable contrast in the +/− allele effect magnitude was observed in some cases, such as the chr 9 MTA, for which N06 (CL0J173-6-8) contributed an allele associated with a +239 kg ha^-1^ effect, whereas N50 (PI 561370) contributed an allele associated with a -326 kg ha^-1^ effect (Figure 2; Table S1). We suggest several possible explanations for inconsistent sign and magnitude of estimated allelic effects among the families. Loci with contrasting effects for yield may be segregating near the same marker in different families due to historical recombination between these loci and markers in founder parents. Also, marker alleles may tag multi-allelic functional haplotypes, most likely in *cis*-regulatory regions, with distinctly different quantitative impacts on the trait (Swinnen *et al.* 2016; Rodríguez-Leal *et al.* 2017). Third, genomic backgrounds could influence expression of *cis*-regulatory regions of MTAs (Rodríguez-Leal *et al.* 2017).

The estimated average allelic effect of MTAs for yield across all families was negative (*i.e.*, -16.7 kg ha^-1^), which was expected given that IA3023 is higher yielding than all but three founders. When averaged by founder class, the mean allelic effect was +3.4 kg ha^-1^ for EL founder families compared to -19.2 kg ha^-1^ and -52.1 kg ha^-1^ for BX and PI founder families, respectively (Figure 2; Table S1).

A key project goal was to identify positive effect alleles for yield in significant MTAs that were present in PI or BX founders, but non-existent, rare, or small in effect in EL founders. No such alleles were detected in any PI founder family (Figure 2, Table S1), but were detected in the BX founder family N38 (LG00-3372), which notably contributed 14 positive yield effect alleles (*vs.* IA3023 allele) – three of which were EL-absent. BX founder N39 (LG04-6000) also was unique for contributing a rare but very large (+157 kg ha^-1^) effect allele on chr 8. These two founder lines were derived from recently introduced germplasm from China. It would be of interest to determine if an estimated yield effect of that size would be maintained were it to be introgressed into IA3023. Several EL founders also could be used for the improvement of IA3023 yield, notably N06 (CL0J173-6-8), N03 (4J105-3-4), and N04 (5M20-2-5-2), which contributed many more positive effect alleles (of often large magnitude) *vs.* few negative effect alleles (of mostly small magnitude).

To identify MTAs for two or more traits that could be either a single multi-trait pleiotropic QTL, or closely linked QTL exhibiting linkage-phased allelic effects, significant MTAs for yield, maturity, height, lodging, or seed weight that clustered within 5 cM of each other were binned (Table 3; Tables S1-S2). Cases in which yield QTL and maturity QTL have coincident map positions are common (Grant *et al.* 2010) and generally a maturity QTL allele conferring later maturity also confers greater yield (Kim *et al.* 2012). Five bins containing yield and maturity MTAs were identified on chrs 9, 10, 13, 16, and 18 (Table 3). In chr 10 bin 2, the large effect MTAs for yield and each of the other traits had a common SNP that mapped within 200 kb of GLYMA.10G221500, which is the cloned maturity gene *E2* (Watanabe *et al.* 2011). Seven NAM families segregated for the late maturity *E2* allele *vs.* the early maturity *e2* allele (Figure S1), which was expected based on a founder parent *E2* allele analysis (Langewisch *et al.* 2014). The *E2* allele for later maturity was associated with greater yield (with one exception), taller plants, more lodging and smaller seed mass compared to the *e2* allele for early maturity (Table S1), an indication of potential multi-trait pleiotropism. Coupling phased positive yield - positive maturity also was evident in chr 9 bin 1 for all founders except one BX and three PI. In the other three bins, positive/negative allele effects for maturity and yield were inconsistently phase-paired, implying separate QTL for each trait. This inconsistency was also the case for five bins with yield and seed mass, four bins with yield and lodging, and three bins with yield and plant height (Table 3, Tables S1-S2). The common SNP-tagged maturity-height MTAs in chr 12 bin 2 and the common SNP-tagged height-lodging MTAs on chr 19 bin 1 exhibited coupling phased allelic effects that were −/− in the former (two exceptions) and +/+ in the latter. Surprisingly, chr 18 bin 3 contained a common SNP that tagged the lodging and seed mass MTAs, and a nearby SNP tagging a yield MTA, and all three exhibited estimates of allelic effects that were consistently positive.

**Table 3:** Significant marker trait associations (MTA) for seed yield (Yd), seed mass (Ms), date of maturity (Mt), plant height (Ht), and lodging (Lg) that were placed in separate bins when the MTA peaks were greater than 5 cM apart. The 21 bins with MTAs for more than one trait are highlighted with gray. See Table S2 for detailed information including interval locations and MTA p-values

Chr	Bin							Chr	Bin						
	1	2	3	4	5	6	7		1	2	3	4	5	6	7
1	Ms	Ms						11	Ms	Yld	Mt	Ms	Yd		
2	Yd	Ms							Mt						
		Mt						12	Ms	Mt	Ht	Mt	Yd		
		Ht								Ht					
3	Mt	Yd	Ldg	Yd	Ht	Ldg	Yd	13	Lg	Ms	Yld	Ht			
	Ht	Ms					Ms				Mt	Lg			
4	Ms	Ldg	Ms					14	Lg	Ms	Mt	Lg			
			Mt					15	Mt	Yd	Ms				
			Ht					16	Yd	Yd					
			Lg						Mt	Ms					
5	Ms	Lg						17	Mt	Ms	Yd	Ms			
6	Ms	Mt	Yd					18	Yld	Ms	Yld				
	Ht								Ms		Ms				
7	Mt	Ht	Ms	Ms	Ms	Ldg	Yd		Lg		Mt				
		Lg									Ht				
8	Mt	Yd									Lg				
	Ht							19	Yd	Yd	Yd	Lg			
9	Yd	Ms	Ms	Ht	Mt				Ht			Ms			
	Ht			Lg					Lg						
10	Ms	Yld	Ht					20	Yd						
		Ms													
		Mt													
		Ht													
		Lg													

To identify candidate genes underlying the yield MTAs, a weighted co-expression network analysis was performed (Schaefer *et al.* 2018). This analysis identifies subsets of genes residing near the significant markers, factoring for both genomic distance and number of genes near the most significant marker (see Methods section). Genes within these subnetworks were tested for significant co-expression by comparing their connectivity “density” to all genes in the MTA subnetwork *vs.* the connectivity of genes in random subnetworks equal in size (via resampled distribution). Fifteen different subnetworks were developed. Genes significant in at least two different subnetworks and displaying strong connectivity with one another were deemed candidate genes for the yield MTAs. Ten such genes were identified, connected in two distinct modules (Table 4; Fig. S6).

**Table 4:** Candidate genes underlying marker trait association (MTA) peaks resulting from co-expression analysis

Module	Candidate gene	MTA chr	MTA position	Pvalue (-log10)	Annotation	Arabidopsis ortholog
1	GLYMA.09G001300	9	106196	10.77	FAR1 DNA-binding domain	AT4G15090
1	GLYMA.06G325300	6	51316639	9.29	Methylenetetrahydrofolate reductase	AT2G44160
1	GLYMA.12G242300	12	40077424	4.21	Dynamin family	AT1G59610
1	GLYMA.12G242500	12	40077424	4.21	Methyltransferase domain	AT1G22800
1	GLYMA.11G106500	11	8106372	3.92	Plasma-membrane choline transporter family protein	AT4G38640
1	GLYMA.03G022900	3	2428982	3.44	SEC7-like guanine nucleotide exchange family protein	AT3G60860
2	GLYMA.10G219500	10	45110885	40.77	RNA-binding protein	AT1G71800
2	GLYMA.16G000500	16	34320	15.04	Ribonucleoprotein-related	AT4G24270
2	GLYMA.13G278500	13	37965132	5.66	Nucleolar GTP-binding family protein	AT1G52980
2	GLYMA.11G245000	11	33885696	3.43	Regulator of chromosome condensation (RCC1)	AT3G26100

The candidate gene GLYMA.09G001300 corresponds to chr 9 bin 1 yield and maturity MTAs that were tagged with a common SNP that maps within 8 kb of this gene (Table 4; Table S1-S2). GLYMA.09G001300 has sequence similarity to a transcription factor that has a role in far-red light response in Arabidopsis (Grant *et al.* 2010; Li *et al.* 2010; Tang *et al.* 2013). It is possible that GLYMA.09G001300 has a similar role in soybean resulting in direct impact on maturity and thus an indirect impact on seed yield. The other candidate genes identified from this analysis had varying annotations that could impact yield. This includes annotations and characterizations from model species that have implicated functions related to growth and development (GLYMA.06G325300 and GLYMA.12G242300), seed size (GLYMA.12G242500), and cell size (GLYMA.11G106500) (Favery *et al.* 2004; Liu *et al.* 2017; Taylor 2011; Xiao *et al.* 2006). However, additional research is needed to determine the yield-related roles of these genes.

This NAM population was previously used to study the development of canopy coverage (Xavier *et al.* 2017) by testing the full NAM population in two environments and part of the population in a third environment. The two environments that the full population was evaluated are included in the dataset analyzed in the current study. Six significant MTA regions for canopy coverage were identified in the Xavier *et al.* study and we found significant MTAs in two of these regions. The two regions included the interval on chr 10 where *E2* was mapped and we identified MTAs for all traits in this region. We also identified MTAs in the chr 19 interval that Xavier *et al.* mapped a major MTA for canopy coverage. In this interval, we mapped MTAs for yield, plant height and lodging (Table S1) and this yield effect was consistent with what Xavier *et al.* observed. This provides confirmatory evidence that the previously mapped major MTAs for canopy coverage may have an impact on agronomic traits across a broad range of environments and thus may be useful in yield improvement.

Seed yield is the most important trait for most field crops and this is the first study that we are aware of that uses a NAM population designed to map MTAs for this trait. We identified between 15 and 29 MTAs for the five measured traits, confirming the multi-genic inheritance of each. Both positive and negative effect alleles relative to the high yielding common parent IA3023 were discovered, and two BX founders with exotic ancestry contributed positive alleles that were rare or not existent in EL founders. This NAM population of inbred lines will be an important resource for future soybean research, including validating MTAs observed in this study and using these results to develop models to improve selection methods.
